# Acquisition of a Joystick-Operated Video Task by Pigs (*Sus scrofa*)

**DOI:** 10.3389/fpsyg.2021.631755

**Published:** 2021-02-11

**Authors:** Candace C. Croney, Sarah T. Boysen

**Affiliations:** ^1^Department of Comparative Pathobiology and Animal Science, Center for Animal Welfare Science, Purdue University, West Lafayette, IN, United States; ^2^Comparative Cognition Project, Sunbury, OH, United States

**Keywords:** animal cognition, pigs, animal learning, video tasks with animals, animal behavior

## Abstract

The ability of two Panepinto micro pigs and two Yorkshire pigs (*Sus scrofa*) to acquire a joystick-operated video-game task was investigated. Subjects were trained to manipulate a joystick that controlled movement of a cursor displayed on a computer monitor. The pigs were required to move the cursor to make contact with three-, two-, or one-walled targets randomly allocated for position on the monitor, and a reward was provided if the cursor collided with a target. The video-task acquisition required conceptual understanding of the task, as well as skilled motor performance. Terminal performance revealed that all pigs were significantly above chance on first attempts to contact one-walled targets (*p* < 0.05). These results indicate that despite dexterity and visual constraints, pigs have the capacity to acquire a joystick-operated video-game task. Limitations in the joystick methodology suggest that future studies of the cognitive capacities of pigs and other domestic species may benefit from the use of touchscreens or other advanced computer-interfaced technology.

## Introduction

Cognitive processes, such as memory, attention, and conceptualization permit animals to demonstrate adaptive behavior in complex, dynamic environments (Wasserman, 1993). These processes have been investigated in laboratory animal species, including non-human primates, rats, and pigeons, among other species, but have yet to be fully explored in farm animals (Curtis and Stricklin, 1991; Duncan and Petherick, 1991). Over the past 2 decades, however, investigations of farm animal cognition have significantly increased, in part because of their implications for ethical obligations toward them, as well as for decisions relating to their production, care, and management (Croney et al., 2004; Mendl and Paul, 2004; Birch, 2018; Franks, 2018; Nawroth et al., 2019). Much of the existing literature on farm animal cognition has focused on the abilities of pigs (*Sus scrofa*; for reviews, see Held et al., 2002; Gieling et al., 2011; Marino and Colvin, 2015), although emerging studies have been conducted recently with horses (e.g., Brubaker and Udell, 2016), goats (Briefer et al., 2014), and sheep (Kendrick et al., 2001; Doyle et al., 2013; McBride and Morton, 2018).

Very early studies conducted by Yerkes and Coburn (1915) gave some indication of the pig’s capacity for complex learning. They found that pigs could solve multiple-choice problems presented in arrays of 2–9 boxes requiring them to: (1) select the first box on the right; (2) select the second box on the left; and (3) alternate between selecting first, the box on the right, then on the left. Later studies of cognitive capacities of domestic pigs indicated that they are capable of operant learning to obtain light (Baldwin and Meese, 1977), produce additional heat for their enclosure (Curtis and Morris, 1982; Morrison et al., 1987), and acquire feed (Croney, 1999). They also are capable of spatial learning (Mendl et al., 1997; Laughlin et al., 1999), although it should be noted that disturbances occurring during cognitive tasks have been shown to impair their performance.

Additionally, pigs have also demonstrated the capacity for discrimination and reversal learning (e.g., Klopfer, 1966; Cerbulis, 1994; Maney, 1998). For example, investigation of Klopfer (1966) of the pig’s ability to learn brightness, color, and spatial discriminations, as well as reversal learning, suggested that they could discriminate based on brightness and color, but only if spatial biases were not permitted to develop in response to feeding. Klopfer (1966) also reported that pigs could acquire spatial (left or right) discrimination learning as well as reversal learning. In addition, Maney (1998) documented that pigs could discriminate between the size, shape, and luminance of objects, while Cerbulis (1994) found that pigs could respond discriminatively to human verbal and gestural commands of novel action-object combinations. Further, Croney et al. (2003) found that pigs could perform visual and olfactory discriminations successfully to locate a food resource in a novel environment, while Murphy et al. (2013) reported that pigs could learn tonal discriminations. Broom et al. (2009) found that pigs could use mirror images to locate food hidden outside of their line of sight. Collectively, these results provide evidence that pigs have the capacity to learn fairly complex novel tasks, and thus might be amenable to testing using alternative paradigms for exploring their cognitive capacities.

Computerized video-game tasks have provided an innovative means of investigating animal cognition using a variety of test subjects, from primates to pigeons (Wright et al., 1988; Rumbaugh et al., 1989; Richardson et al., 1990; Rumbaugh, 1990; Spetch et al., 1992; Hopkins et al., 1996; Markham et al., 1996; Leighty and Fragaszy, 2003). These approaches permit control of the exact temporal and spatial parameters of an animal’s responses, and investigators can obtain greater stimulus flexibility because of the relatively unlimited number of visual stimuli that can be generated and presented. This is especially useful for tasks that require large numbers of novel stimuli to test how an animal learns new information over time. Similarly, joystick-operated video-game tasks require the subject to use a joystick to move a cursor until it makes contact with a target on the screen (Rumbaugh et al., 1989). Two characteristics are required for successful task completion. First, the animal must have sufficient motor skills to be able to manipulate a joystick. Secondly, the animal must have the cognitive ability to learn that joystick movements control cursor movement, and that the collision of cursor and target is followed by a reward.

Video-task acquisition has also been demonstrated in a range of primates, including rhesus monkeys, baboons, gorillas, and chimpanzees (Rumbaugh et al., 1989; Richardson et al., 1990; Lincoln et al., 1994; Hopkins et al., 1996). Computerized tasks have been used to test spatial memory in pigeons (Spetch et al., 1992) and matching-to-sample in pigeons (Wright et al., 1988), as well as operant conditioning and visual discrimination in rats (Markham et al., 1996). Because computerized tasks provide a more objective means of testing some types of cognitive processing in animals, allow for a wider variety of test stimuli, and permit precise control of stimuli and recording of responses, the current study was undertaken to explore the pig’s ability to acquire a joystick-operated video-game task and evaluate the usefulness of this technology for further investigations of their cognitive abilities.

## Materials and Methods

### Subjects

Two Yorkshire barrows (castrated male pigs) and two Panepinto micro pig barrows served as subjects. The animals were maintained in an indoor facility on the Pennsylvania State University campus. The Yorkshire barrows (60 and 63 kg, respectively, at the beginning of the study) were both 3 months old, and housed together in an indoor pen measuring 1.83 m × 2.3 m. The Panepinto micro pigs (43 and 50 kg, respectively) were both 24 months old and also were housed together in an indoor pen measuring 1.26 m × 2.3 m. All pigs were maintained on cement floors covered with rubber stall mats. The Yorkshires were fed a balanced, fortified, corn-soy diet *ad libitum* daily. The Panepinto micro pigs were fed 1–1.5% body weight (kg) mini-pig diet (Lab mini-pig HF grower diet 5 L80, PMI Feeds Inc., St. Louis, MO). Continuous access to an operant waterer was provided for all pigs, and all subjects were maintained on a 12/12 h light/dark cycle.

### Apparatus

**Figure 1:**
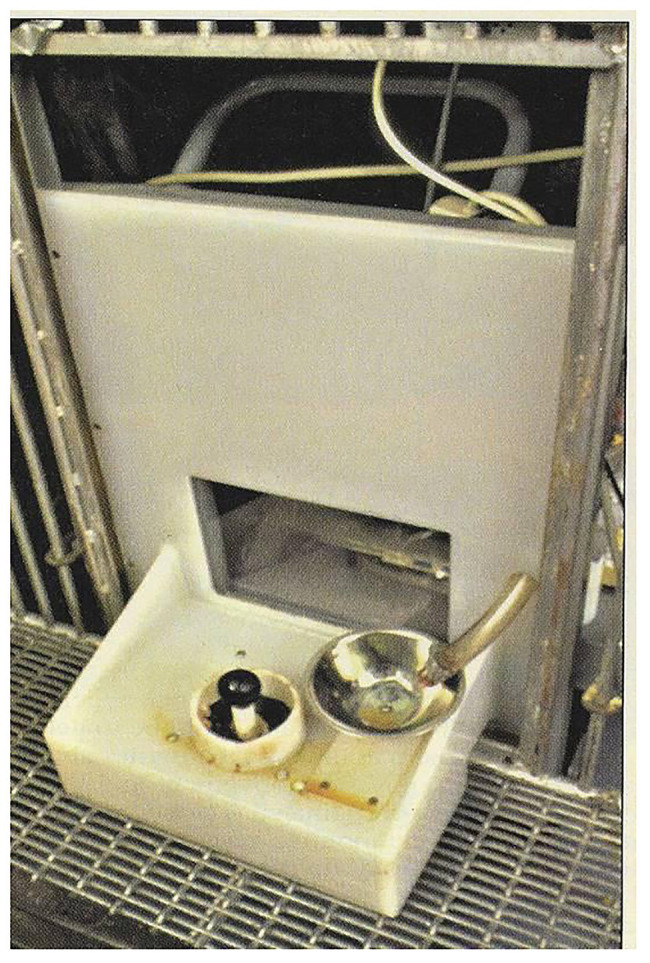
Joystick apparatus for testing pigs.

To determine the ability of pigs to acquire a complex visuo-spatial task using a joystick, the NASA/LRC computerized test system was used (Rumbaugh et al., 1989). The experimental apparatus consisted of an IBM 386 personal computer with a 33-cm color monitor, positioned behind a transparent Lexan window, with a modified 11-cm analog joystick shaft (Flight Pro analog joystick, model SV-215), attached to a 4.5 cm diameter gear shift knob, and a Med Associates SG-601 automatic pellet reward dispenser. The apparatus was located in an elevated testing pen (0.77 m high × 2.14 m wide × 1.50 m long) that was constructed of metal gates and woven wire flooring. A ramp constructed of 0.017 m plywood measuring 1.22 m × 0.61 m (0.42 m high at top and angled at approximately 45 degrees) and a guillotine door made from 0.007 m plywood (0.84 m × 0.53 m) allowed access to the test pen (Figure 1).

Prior to the experiment, the focal length of the pigs was determined by lens refraction conducted by an optometrist to find the best position for the computer monitor (see Michaels, 1975). All pigs were found to be far-sighted, with each subject determined to be between +1 and +2 diopters hyperopic. To accommodate their visual limitations, the computer monitor was positioned approximately 45 cm away from the subjects’ eyes when they were using the joystick.

### Pre-training

**Figure 2:**
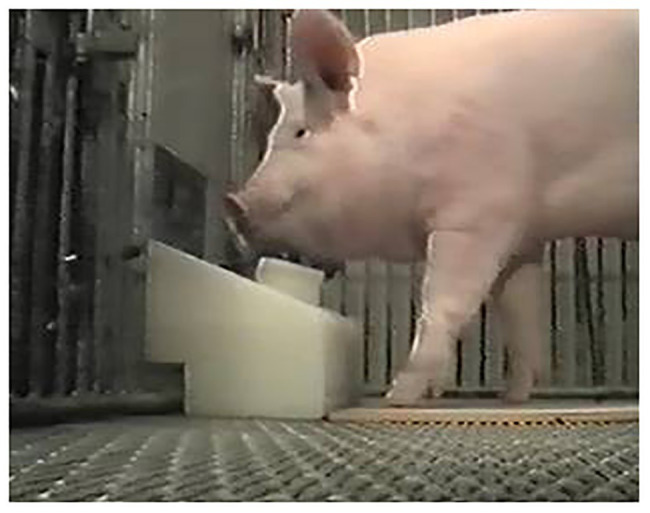
Pig subject using joystick during testing with SIDE Task.

A mock joystick apparatus was constructed for pre-training purposes, consisting of a black plastic gear shift knob (4.5 cm diameter) mounted on a spring, and attached to a plywood base 1.8 cm thick × 28.3 cm long × 22.5 cm wide. Sections of 10 cm PVC pipe were cut and fastened together to form a tube which delivered food rewards into a PVC cup (10 cm diameter) attached to the plywood base, approximately 10 cm from the base of the mock joystick. The pigs were shaped to approach the joystick and manipulate it with their snouts. Each time they approached the mock joystick, they were rewarded with a dog food pellet as the handler gave the command “Joystick.” Eventually, the pigs were rewarded only when they approached and manipulated the joystick with a verbal command. Shaping sessions lasted approximately 10 min for each subject, and were conducted once daily, 5 days per week, until the pigs manipulated the joystick consistently on command. The mock joystick training was conducted for 2 weeks, after which the pigs reliably performed the behavior (Figure 2).

After the 2-week training for joystick manipulation, the pigs were shaped to watch the computer monitor when it was positioned in the experimental testing apparatus, as previously described. All subjects were tested individually, and when in position on the elevated apparatus, were given the command, “Watch the screen.” When the pigs oriented toward the window in front of the computer screen, a reward was dispensed. When they were able to perform this behavior consistently, the command “Watch the screen” was paired with the command “Joystick.” The pigs were reinforced immediately for attending to the computer monitor, and then manipulating the modified joystick.

### Side Training

After pre-training, the pigs were trained to perform a rudimentary joystick-operated video game task. The task, referred to as the SIDE task (Hopkins et al., 1996), began with a computer-generated 2.5 cm blue border around the inside edges of the computer screen which created four target walls. A white 2-cm circular cursor appeared in the center of the screen. Movement of the joystick in any direction caused the cursor to move at a rate of 8 cm/s. The pigs were trained to move the joystick to contact one of the target walls. Contact resulted in auditory feedback from a speaker (computer-generated “bloop” sound) and the delivery of a food reward (dog food pellet). An experimenter stood outside the test pen and provided the pigs with verbal and tactile reinforcement after each successful trial. Successful and unsuccessful attempts to contact target walls were recorded by the SIDE software program, and targets were randomly assigned to positions on the screen (above, below, and left or right of center). A titrated version of the SIDE task based on response latency was utilized, so that as a subject’s performance improved or declined, task difficulty increased or decreased, accordingly. For example, as subjects completed a number of trials (usually five or six) within a fixed period (less than 10 s) successfully, the number of target walls was successively decreased from four target walls, to three walls, to two walls, and finally to one wall. After successful performance on the one-walled condition, the target size was successively decreased to create partial walls of different sizes (from 16 cm to 6 cm, then to 2.5 cm). Alternatively, if the subject failed to complete trials within the allotted time, the number (or size) of target walls was successively increased. Subjects were tested once daily, 5 days per week, for 12 weeks. Data were analyzed from all sessions in which pigs completed a minimum of 15 trials.

### Revised Training

After approximately 4 months of training with the Panepinto micro pigs, we observed that because of the titration of the task, the pigs were completing a disproportionate number of four- and three-walled targets during sessions and were therefore making little progress on two- and one-walled targets. To correct this, the pigs were now required to complete a minimum of 15 two- and one-walled targets during each session. The Yorkshire pigs had been terminated from the experiment prior to this, and thus they were tested using only the titrated version of the task described above.

### Statistical Analyses

Each subject’s percentage of correct responses contacting a target wall with the first cursor movement was recorded for three-, two-, and one-walled conditions and for each of the target positions (above, below, left, and right of screen center). Because performance on four-walled targets was always 100% provided the subject completed contact, four-walled target performance was not analyzed. Performance on two- and one-walled targets was of special interest because success on these categories was more indicative of the pigs’ ability to acquire the concept underlying the task, in addition to the required motor skills. Statistical analysis was performed in R version 3.6.2. Binomial testing was used to compare each subject’s percentage of correct first cursor movements during their terminal performance (final block of 50) to the expected probability of success due to chance (i.e., three-walled test = 75%, two-walled test = 50%, and one-walled test = 25%).

According to Hopkins et al. (1996), in which primates’ abilities to acquire the SIDE task were evaluated, criterion for demonstrating motoric skill acquisition was completion of a block of 100 trials, with at least 50% of the trials consisting of partial 1-wall targets. These investigators considered conceptual understanding of the task established when over 90% of a block of 100 trials consisted of 1-wall or partial-wall trials. At this performance level, they reasoned that the subjects understood the discriminative requirements of the SIDE task since they could move the cursor to the correct target position on a consistent basis (Hopkins et al., 1996). These are relatively strict criteria that require good dexterity by the subjects.

## Results

### Yorkshire Pigs: Number of Target Walls

**Figure 3:**
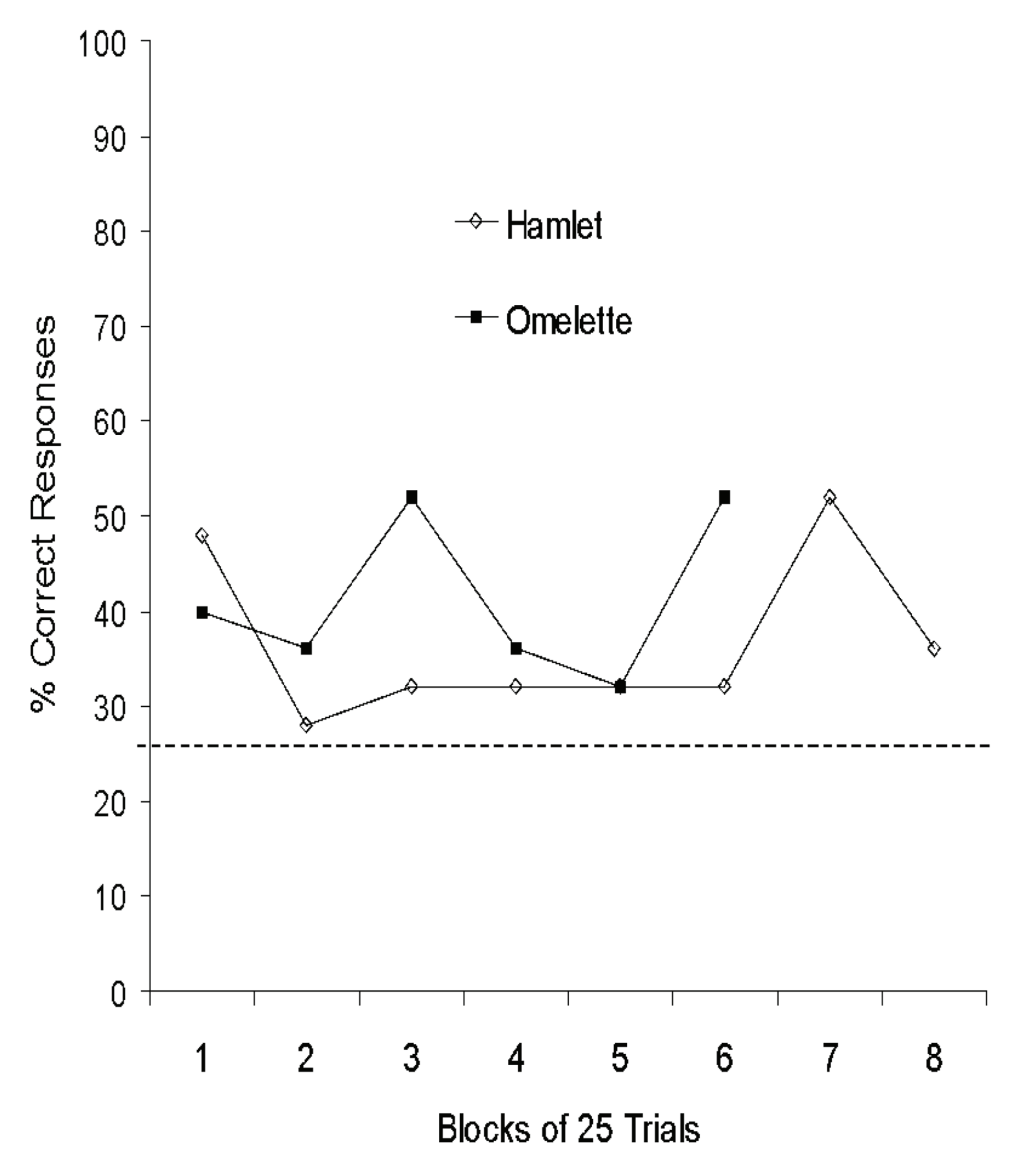
Performance of Yorkshire pigs on one-walled targets collapsed across target position. Horizontal dashed line indicates chance performance.

Analyses of terminal performance (last block of 50 trials) showed that neither of the Yorkshire pigs (Hamlet and Omelet) achieved significant performance on three-walled targets (*p* > 0.05; Table 1). On two-walled targets, both Yorkshires were above chance with 78 and 70% correct responses, respectively (*p* < 0.001). Both pigs performed above chance on one-walled targets when collapsed across target wall position (Hamlet: 48%; Omelet: 42%; *χ*^2^ = *p* < 0.01). The Yorkshire pigs’ performance on one-walled targets over time is presented in Figure 3.

**Table 1:** Terminal performance of Yorkshire pigs on SIDE task.

Subject	Category	*n*	%correct responses	%Chance	*χ*^2^	*p*
Hamlet						
	Left	10	20	25	1.30	n.s.
	Right	15	27	25	0.21	n.s.
	Above	14	71	25	112.80	^****^
	Below	13	69	25	103.20	^****^

	3-wall	50	80	75	1.33	n.s.
	2-wall	50	78	50	31.36	^****^
	1-wall	50	48	25	28.21	^****^
Omelet						
	Left	15	27	25	0.21	n.s.
	Right^a^	–	–	25	–	–
	Above	15	27	25	0.21	n.s.
	Below	16	69	25	103.00	^****^

	3-wall	50	74	75	0.053	n.s.
	2-wall	50	70	50	16.00	^****^
	1-wall	50	42	25	15.41	^****^

Performance on target positions (left, right, above, and below) was analyzed and presented as a function of the last 50 one-walled trials completed.

a Due to injury, Omelet was unable to continue training long enough to complete the minimum number of five trials in this category.

**** indicates significance at the 0.001 level.

#### Target Position

Performance on the target position was analyzed for one-walled targets (see Table 1). Both pigs demonstrated response biases, generally performing better on vertical plane (up, down) movements than horizontal plane (right, left) movements. Hamlet was 71% correct on one-walled targets when the target was located above (*p* < 0.001), and 69% when the target was located below screen center (*p* < 0.001). Omelet was 27% (*p* = 0.645) and 69% (*p* < 0.001) correct on one-walled targets located above and below screen center, respectively.

#### Learning Curves

Terminal performance (last block of 50 trials) was compared to performance on the first block of 50 trials for each category of number of walls (3-, 2-, and 1-walls) and on target positions. Omelet showed no significant improvement on three- or one-walled targets over time, but did improve on two-walled targets (*p* < 0.05). Hamlet likewise improved on two-walled targets (*p* < 0.025), but not on three-walled targets. Surprisingly, his performance on three-walled targets actually declined toward the end of the experiment (*p* > 0.10). Hamlet’s performance on one-walled targets also did not improve significantly.

After 12 weeks of training, Hamlet and Omelet were terminated from the experiment because they had grown too large to stand long enough to complete sessions, and also no longer fit within the constraints of the test pen.

### Panepinto Micro Pigs: Number of Target Walls

The Panepinto micro pigs’ (Ebony and Ivory) terminal performance (last block of 50 trials) was analyzed (Table 2).

**Table 2:** Terminal performance of Panepinto micro pigs on SIDE task.

Subject	Category	*n*	% correct responses	%Chance	*χ*^2^	*p*
Ebony						
	Left	11	100	25	300.00	^****^
	Right	11	0^a^	25	33.33	^****^
	Above	13	61.5	25	82.12	^****^
	Below	15	20^a^	25	1.33	n.s.

	3-wall	50	84	75	4.32	^*^
	2-wall	50	56	50	1.44	n.s.
	1-wall	50	34	25	4.32	^*^
Ivory						
	Left	12	67	25	94.08	^****^
	Right	13	85	25	192.00	^****^
	Above	13	69	25	103.25	^****^
	Below	12	42	25	15.14	^****^

	3-wall	50	84	75	4.32	^*^
	2-wall	50	68	50	12.56	^****^
	1-wall	50	76	25	138.72	^****^

Performance on target positions (left, right, above, and below) was analyzed and presented as a function of the last 50 one-walled trials completed.

a Performance was below chance.

* indicates significance at a 95% confidence level.

**** indicates significance at the 0.001 level.

**Figure 4:**
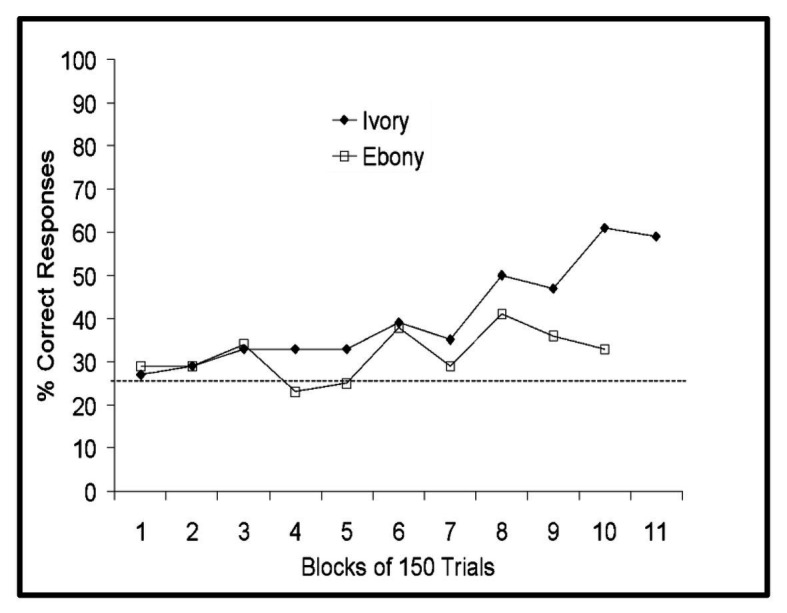
Performance of Panepinto micro pigs on one-walled targets collapsed across the target position. Horizontal dashed line indicates chance performance.

On three-walled targets, both pigs were more successful than would be expected by chance (Ebony: 84%, *p* = 0.038; Ivory: 84%, *p* = 0.038). However, while Ivory was above chance when presented with the two-walled task (68%, *p* < 0.001), Ebony was not (56%, *p* = 0.271). Furthermore, Ebony performed only marginally better than expected by chance when presented with one-walled targets (34%, *p* = 0.049), while Ivory was 76% correct on one-walled targets (*p* < 0.001). The micro pigs’ performance on one-walled targets over time is presented in Figure 4.

#### Target Position

The micro pigs’ performance on target position was also analyzed as a function of one-walled targets (see Table 2). Like the Yorkshire pigs, Ebony and Ivory demonstrated response biases, particularly on one-walled targets. However, unlike the Yorkshires, they generally performed better on horizontal plane (right, left) movements than vertical plane (up, down) movements. Ebony showed a strong bias for one-walled targets positioned to the left of screen center, while Ivory’s bias was for right-sided targets. Ebony’s performance was significantly below chance on one-walled targets positioned to the right of the screen (0%; *p* < 0.001) and below chance when the target was located at the bottom of the screen (20%; *p* = 0.292). Ivory’s performance differed from that of the other three pigs in that the disparity in his performance based on target position was relatively small. In fact, he was the only subject to perform well above chance on all positions (*p* < 0.001).

#### Learning Curves

The micro pigs’ terminal performance (last block of 50 trials) was compared to initial performance (first block of 50 trials) for each category. Ivory’s performance increased significantly for all target positions and number of walls (*p* < 0.001) except the three-walled condition. Ebony improved on targets to the left and top of the screen center (*p* < 0.001) and on three- and two-walled targets (*p* < 0.05, *p* < 0.001, respectively). After 15 months on the SIDE task, Ebony and Ivory’s training was terminated.

Due to limitations of the version of SIDE task software utilized, it was not feasible to electronically extract data in a manner that would have permitted accurate, detailed analyses of error patterns for each individual. Future programming for similar or related tasks, indeed, for any species tested using advanced technology, should be sure to include the potential for such evaluation, as the error patterns observed and identified during some facets of the experiment may provide valuable information as to how information processing and physical manipulation of the joystick (or other manipulanda) subserve the animals’ resulting performance.

## Discussion

Overall, all pigs performed significantly above chance on one-walled targets, which indicates that, to some extent, all acquired the association between the joystick and cursor movement. That the pigs achieved the level of success they did on a task that was significantly outside their normal frame of reference in itself remarkable, and indicative of their behavioral and cognitive flexibility. Their high level of social motivation to perform the task was also noteworthy. Although food rewards associated with the task were likely a motivating factor, the social contact the pigs experienced with their trainer also appeared to be very important. Occasionally, during some sessions, equipment failures resulted in non-reward following correct responses. On these occasions, the pigs continued to make correct responses when rewarded only with verbal and tactile reinforcement from the experimenter, who was also their primary caretaker. Additionally, during times when the task demands seemed most challenging for the pigs, and resulted in reluctance to perform, only verbal encouragement by the experimenter was effective in resuming training. This may have been due to the strong bond the pigs developed with the experimenter during training, which would support the assertion of Boysen (1992) that the human-animal bond is a crucial element in the success of animals used in studies of comparative cognition.

It should be noted that despite performing above chance on the SIDE task, even the pig that performed best did not approach the level attained by non–human primates that acquired the task after a comparable number of trials (see Hopkins et al., 1996). Indeed, none of the pigs was able to meet the criteria of Hopkins et al. (1996) for demonstrating motoric or conceptual acquisition of the SIDE task. There are several possible explanations for the pigs’ failure to meet they criteria. First, they were established for dexterous primates (rhesus monkeys and chimpanzees); although no clear rationale was provided for their adoption. Thus, it was difficult to know how to adapt those criteria for pigs, taking into account their more limited perceptual and motor capabilities, which clearly differ from primates. For example, the visual demands of the task may have been particularly problematic for the pigs, since we had previously established that all four subjects were far-sighted. As sufficient visual capability is a prerequisite for successful completion of a joystick-operated-video game task, and despite attempts to position the computer monitor appropriately, it is impossible to know how well the pigs were able to see, and subsequently correctly discriminate between targets. Furthermore, because of the positioning of the pigs’ eyes relative to their snouts, they were often forced to watch the screen prior to moving the joystick, and then check their progress after cursor movement was initiated. This artifact of the pigs’ anatomy likely contributed to some of their errors because in order to succeed, they not only needed dexterity and conceptual understanding of the task, but perhaps also short-term or working memory (which is not well understood in pigs) of the target position locations.

In addition, the pigs’ limited dexterity no doubt constrained their performance. Because the joystick-operated video-task paradigm was initially designed for use by non-human primates with great manual dexterity, modifications to the equipment were necessary so that the pigs could use their snouts to manipulate the joystick. However, the pigs’ ability for such manipulation was restricted to their normal range of head and neck movements. This limitation appeared particularly troublesome for the Yorkshire pigs whose larger size also constrained their ability to reposition themselves as needed to contact targets located in the horizontal plane. Thus, it was not surprising that the Yorkshire pigs performed better on vertical plane movements, which are more frequently seen in their normal behavioral repertoire during routine activities such as rooting. In fact, when faced with left or right targets, the Yorkshire subjects were often observed to alter their stance so that they were parallel to the computer screen. This way, they could approach horizontal targets in the same way they did for those in the vertical plane. Because of their small size, the micro pigs were better able to reorient themselves as needed to view the computer monitor and complete horizontal plane movements. This flexibility likely resulted in better performance in both planes and may have contributed to their superior performance compared to the Yorkshire subjects. Ebony and Ivory’s smaller size also enabled them to be maintained in the laboratory for a much longer period for training and testing (15 months) than the Yorkshire pigs. Thus, they were afforded the opportunity to continue training, thereby contributing to their improved performance on the SIDE task. Consequently, their terminal performance was much better than the Yorkshire pigs that were trained for only 10 weeks on the same task.

Additional problems that may have been attributable to dexterity limitations were observed when the pigs were unable to completely move the cursor toward a target wall and finish the trial, simply because of the angle at which the cursor approached the target. On these occasions, the pigs often nosed the joystick to move the cursor back out of the target wall and then altered the angle at which they approached the target. However, in doing so, they sometimes contacted an incorrect wall, resulting in reduced accuracy on their first cursor attempts. Further, when the pigs were unable to make contact with a horizontal target, they often resorted to strategies that allowed them to move the cursor upward, then down into the correct left or right wall. These responses were consistently observed, particularly for Hamlet and Omelet, who systematically responded with a series of movements that resembled an “inverted v” when faced with right or left targets. The resultant asymmetry in the pigs’ performance relative to target position is similar to that observed in rhesus monkeys (Hopkins et al., 1996). In comparing the performance of rhesus monkeys to chimpanzees on the SIDE task, Hopkins et al. (1996) observed that the monkeys had more difficulty responding to horizontal targets, suggesting that their manipulative behavior was less diverse than chimpanzees. This problem may, in part, explain the pigs’ poor performance relative to primates, as their ability to manipulate objects is significantly less dexterous and flexible.

Response biases can often be inevitable when testing animals, and they emerged during testing with the pigs as well. For example, while Ebony, like all of the subjects, showed some level of side bias (left), he did correctly move the cursor to the right numerous times on all but the one-wall task. As previously noted, these trials created the smallest targets for the pigs. Side bias training was instituted for all pigs manually upon observation of biases because although the software titrated to an easier level of task difficulty if a subject made errors consistently, the program’s random generation of target locations did not facilitate training to overcome bias. This intervention was not successful, however. Learning on manual side-bias training with objects or with the joystick with the computer turned off (necessary given the previously mentioned software limitations) did not appear to generalize to the joystick-operated task. A few explanations for this observation are plausible. First, Ebony may simply have been limited in either or both dexterity and the paw/snout/eye-coordination needed to hit right-sided, one-walled targets. It is also possible that because the video-task apparatus was not centered in the pen due to constraints of the testing space, Ebony’s body positioning to complete such tasks may have further constrained his performance given that additional training did not correct the side-bias problem with the joystick, although it was effective on bias correction using objects (Croney, 1999). It is also possible that some degree of instinctive drift may have impacted his and the other pigs’ performance, especially as the tasks became more challenging and rewards for behaviors performed were reduced due to errors.

An alternative explanation for the difference between the pigs’ and primates’ performance that must be considered is that the pigs may have been unable to fully comprehend the concepts required to perform well on the SIDE task. Difficulties with the conceptual component of the task may have been due, in part, to the spatial discontiguity of the stimulus and response. Meyer et al. (1965) suggested that a primate’s learning efficiency might be impaired when the hand used to execute a response was placed in an area distant from the location of the discriminative stimuli. A similar rationale may have been a factor for the pigs, since the movement of their snouts was some distance from the images displayed on the monitor, and the lateral placement of their eyes may have contributed to a cognitive disconnect between their movements and the resulting changes appearing on the screen.

In addition to the difficulties posed by limited dexterity and vision, several methodological factors may also have impeded the pigs’ performance on the SIDE task. First, because a protocol for testing pigs using the joystick-operated video-game task paradigm had not previously been established, the methods used in the current experiment were exploratory. As such, some changes in procedures and equipment were necessary during the experiment to correct concerns as they emerged. For instance, early design flaws in the joystick apparatus were detected and required correction. Initially, the protective welded plastic area surrounding the joystick was too high and impeded movement of the joystick in all directions. In addition, positioning of the feed delivery tube attached to the automatic dispenser sometimes resulted in failure to deliver rewards to the pigs after correct responses early in training and required correcting. This delay in reinforcement following a correct response may have impeded the animals’ initial learning. Finally, the test pen was designed so that the joystick apparatus was positioned approximately 0.04 m away from the right side of the pen. This initial positioning proved to be significant in that it restricted the pigs’ abilities to stand or move to the right of the joystick.

Initial training procedures also proved to be problematic. One problem in the training process was that the pigs were allowed to work at their own pace, which resulted in a large set of data consisting primarily of four- and three-sided tasks. After the protocol was amended to require performance of a minimum number of two- and one-walled targets during each session, improved performance on these conditions was observed. However, the Yorkshire pigs had been terminated from testing by the time procedures were revised, and thus did not benefit from the revision. Moreover, this change in training made it extremely difficult for the micro pigs to achieve stringent criteria of Hopkins et al. (1996) for all facets of task acquisition.

Taken together, the failure of all subjects to meet the criteria for SIDE task acquisition may reflect the limitations first imposed by procedural methodology issues, and visual and motor skill limitations, rather than learning deficits. Although their performance was limited compared to primates tested, that they were able to perform as successfully as they did on one-walled targets suggests they acquired some important aspects of the task demands. However, it is impossible to determine to what extent their ability to demonstrate conceptual understanding of the SIDE task may have been constrained by their perceptual and motor capacities. Nonetheless, evaluation of their terminal test results showed that all pigs improved their performance with respect to the various target positions. This improvement was particularly noteworthy for the Yorkshire pigs (Hamlet and Omelet), who completed only a few 100 trials in their 10 weeks of training on the task. Furthermore, the high level of performance attained by one of the micro pigs (Ivory), regardless of target position or number of walls, strongly suggests some level of conceptual acquisition of the task.

In summary, the results of the present study underscore the importance of understanding the basic perceptual and motor capabilities of a species prior to developing appropriate methods of testing their cognitive abilities. While the joystick-operated video-game paradigm has proven suitable for testing several species, including monkeys, pigeons, and chimpanzees (Rumbaugh et al., 1989; Washburn et al., 1990; Spetch et al., 1992; Hopkins et al., 1996), it is not optimal for testing the cognitive abilities of pigs, as their performance was clearly hindered by dexterity limitations and visual constraints. Thorough investigations of the pig’s visual and motor capabilities are necessary before their cognitive abilities can be adequately assessed using this or any type of technology. Use of a computer touch screen may better address the problem of limited dexterity and would likely provide a more viable alternative in future computer-interfaced studies of the cognitive abilities of pigs.

## Data Availability Statement

The raw data supporting the conclusions of this article will be made available by the authors, without undue reservation.

## Ethics Statement

The animal study was reviewed and approved by the Pennsylvania State University, State College, Pennsylvania, United States.

## Author Contributions

All data collection was performed by CC. Data analyses were conducted by CC and SB. Contributions to the experimental design by SB. Writing and editing of the paper was conducted by CC and SB. Both authors contributed to the article and approved the submitted version.

### Conflict of Interest

The authors declare that the research was conducted in the absence of any commercial or financial relationships that could be construed as a potential conflict of interest.
